# Facilitators and Barriers to Receiving Palliative Care in People with Kidney Disease: Predictive Factors from an International Nursing Perspective

**DOI:** 10.3390/nursrep14010018

**Published:** 2024-01-22

**Authors:** Ilaria de Barbieri, Veronica Strini, Helen Noble, Claire Carswell, Marco Bruno Luigi Rocchi, Davide Sisti

**Affiliations:** 1 Woman’s and Child’s Health Department, Padua University Hospital, 45128 Padova, Italy; ilaria.debarbieri@aopd.veneto.it; 2 Clinical Research Unit, Padua University Hospital, 45128 Padova, Italy; 3 School of Nursing and Midwifery, Queens University Belfast, Belfast BT9 7BL, UK; helen.noble@qub.ac.uk (H.N.); c.carswell@qub.ac.uk (C.C.); 4 Department of Biomolecular Sciences, Service of Biostatistics, University of Urbino, 61029 Urbino, Italy; marco.rocchi@uniurb.it (M.B.L.R.); davide.sisti@uniurb.it (D.S.)

**Keywords:** conservative management, end-stage kidney disease, nurse, palliative care, Delphi study

## Abstract

Background: Palliative care (PC) focuses on relieving pain and difficult symptoms rather than treating disease or delaying its progress. Palliative care views death as a natural process and allows patients to live the last phase of their existence in the best possible way, encouraging them to express their opinions and wishes for a good death. Interventions are advocated to control symptoms and distress and promote wellbeing and social functioning. A multidisciplinary approach to support patients receiving palliative care is encouraged. Objective: The aims of this study were to investigate the facilitators and barriers to PC in people with kidney disease from a nursing perspective and to explore predictive factors associated with nurse-perceived facilitators and barriers to PC in people with kidney disease. Design: This study is a survey that adopted a questionnaire created in 2021 with Delphi methology, which included 73 statements divided into 37 facilitators and 36 barriers to PC in patients with kidney disease, to be scored using a Likert scale. Participants and Measurements: Participants were obtained through the membership database of the European Dialysis and Transplant Nurses Association/European Renal Care Association (EDTNA/ERCA) of 2020. Inclusion criteria included being registered as a nurse, an EDTNA/ERCA member and understanding of the English language. The questionnaire was sent via email. Results: Three profiles of respondents were found: the first group was characterized by the highest agreement percentages of facilitators and with an average value of 53.7% in barriers; the second was characterized by a lower endorsement of facilitators and similar agreement to the first group for barriers; the third group had a high probability (>80%) of items endorsing both barriers and facilitators. Predictive variables were significantly associated with “Years in nephrology” and “macro geographic area”. Conclusions: This study demonstrates variation in PC practice across Europe. Some professionals identified fewer barriers to PC and appeared more confident when dealing with difficult situations in a patient’s care pathway, while others identified more barriers as obstacles to the implementation of adequate treatment. The number of years of nephrology experience and the geographical area of origin predicted how nurses would respond. This study was not registered.

## 1. Introduction

The aim of palliative care (PC) is to improve the quality of life of patients, families and caregivers by resolving problems associated with the disease. This involves the treatment of pain, as well as social and spiritual problems associated with the disease [1].

PC is defined as a treatment that aims to improve the quality of life of people suffering from chronic diseases, alleviating pain and symptoms rather than intervening directly in the treatment of the disease or delaying its progress [2]. Medical assistance cannot ignore good management of the psychosocial and spiritual sphere, correct coordination between services and the creation of a support system to help the patient and his family face the moment of death [1].

The annual mortality rate of dialysis patients is approximately 20–25% in the general population, reaching 50% in frail elderly patients [3]. The mental and physical burden of patients with advanced chronic kidney disease (CKD) is similar to that found in cancer patients [4].

Nephrologists are becoming more aware that PC is not only the management of the illness at the end of life but a pathway that can be applied to patients to offer support along a trajectory toward the end of life [5]. Grubbs et al. [6] state that palliative care is particularly suitable for dialysis patients with a life expectancy of less than one year. Holley [7] suggests that PC in dialysis requires an interdisciplinary approach, including management of pain and other symptoms, advanced care planning, communication with the patient and taking into account ethical issues regarding the end of life. Therefore, the ability to communicate with patients and their families is an indispensable skill for effectively sharing information. Good information allows the patient to be able to repeat what was said and understand [4]; this process is the basis of shared decision making (SDM). This process must be implemented before starting dialysis, enabling patients to understand the benefits, risks and alternatives to dialysis [8].

Patients with end-stage kidney disease (ESKD) may be offered dialysis, renal transplantation or conservative management [9,10]. Conservative management (CM) is a planned patient-centered approach indicated for patients unlikely to benefit from dialysis or who choose not to dialyze [4].

Withdrawal from dialysis treatment usually signifies the end of life of a patient with a predictable death and the end of long-term relationships with staff [11]. The communication of different choices of treatment places renal nurses in a unique situation [12].

End-of-life care planning is highly relevant for people with ESKD, their families and health professionals. The current integration of PC into the plan of care is influenced by the values, beliefs and knowledge of the health professionals [9,13,14].

The nephrology nurse is often the first person who hears the patient’s request to continue or stop renal replacement therapy [15]. Some nurses may express frustration if a patient’s wishes are overlooked by physicians [16] and many wish to honor the patient’s wishes [17].

A lack of training has been identified as a factor creating variation in the management of end-of-life care [7]. Medical and nursing teams affirm they feel distressed about giving the patient a hand in the decision-making process about withdrawing from dialysis or continuing life-prolonging treatment, sometimes without a complete discussion and understanding of the possibilities available to them [11,16,18]. Medical and nursing curricula should include theoretical and practical teaching to enable healthcare practitioners to support patients and their families as death approaches [19]. 

In recent years, programs have been implemented in the fields of palliative care but greater focus should be given to these programs to help patients, families and medical staff prepare for impending death.

## 2. Literature Review

Advanced care planning (ACP) and SDM are part of the standard of care for cancer but not for nephrology [20]. Nephrologists often face difficult conversations, such as giving poor prognosis, talking about the onset or withdrawal from dialysis or about end-of-life care [4]. Dialysis, in particular, can extend life but it might not improve quality of life [20]. In this case, clear communication regarding prognosis will impact a patient’s choice of treatment.

CM can facilitate the integration of the patient’s lifestyle, family and community into the treatment plan. For some, however, CM is not a choice but the only option if dialysis is financially prohibitive [12]. Palliative care supports the patient at the end of life so that he can adhere to his own beliefs, values, culture and religion, living this phase as a natural process and in the best possible way [13,15].

However, this approach is not always well accepted in the renal context: Sturgill and Bear [21] underline how the models of care still present for the CKD population are underdeveloped in some realities, such as the US context, while in others, they are better developed (as in Australia, where patients are followed by multidisciplinary teams that include both nephrology and palliative specialists).

As suggested by Hermann’s [22] study, ESKD patients and cancer patients in the final stages of life have spiritual needs, such as the need for hope and understanding of the meaning of life. In such cases, there are other figures alongside healthcare professionals who can be of support such as counselors, supportive services and hospital clerics [23].

Little is currently known about the facilitators and barriers to PC in the renal context and there are no data at a European level from a nursing perspective [12]. De Barbieri et al. [12] used the Delphi methodology to create a questionnaire to explore the views of nurses on barriers and facilitators to PC in patients with kidney disease. The questionnaire was evaluated by experts in the renal specialty across Europe and subsequently included 73 statements, including 37 facilitators and 36 barriers, which rank your own agreement on a 4-point Likert scale.

The aims of this study were to use de Barbieri et al. [12]’s questionnaire:

- to investigate the facilitators and barriers to PC in people with kidney disease from the perspective of renal nurses;
- to explore predictive factors associated with these perceived facilitators and barriers.

## 3. Materials and Methods

The study adopted the questionnaire created with the Delphi technique in the study by de Barbieri et al. [12]. The questionnaire included 73 statements divided into two domains: 37 facilitators and 36 barriers to PC in patients with kidney disease (Supplementary Material).

### 3.1. Participants

Participants were obtained through the membership database of the European Dialysis and Transplant Nurses Association/European Renal Care Association (EDTNA/ERCA) of 2020. The invitation to participate in the study was sent by email to all active members of the association EDTNA/ERCA, sending together an explanatory statement regarding the study and a link to complete the survey.

The questionnaire was sent to 612 contacts.

Participants had to be qualified as nurses, be members of EDTNA/ERCA and have a good knowledge of the English language. There is no specific recommendation for experience in PC or conservative care, although it may be favorable.

Nurses are recognized worldwide as the HCPs who spend the most time with patients and have the most contact with people affected by disease and their families [24]. Nurses were likely to have experienced barriers and facilitators in the palliative care setting and it was seen as important that their views were represented.

The following variables were collected: gender, age, years of clinical practice, years of practice in nephrology, working organization (public/private), believer (no; yes, non-practicing; yes, practicing), religion (atheism; Christian protestant; Christian catholic; Christian orthodox; Islamic) and macro-geographic area (northern Europe, southern Europe, central Europe, eastern Europe, non-European countries).

### 3.2. Ethical Consideration

The data collected were handled in accordance with the EU General Data Protection Regulation (GDPR) (anonymous data). The study, not involving analysis of sensitive data, did not require submission by the local CE, in line with the national legislation of the coordinating state.

An invitation email was initially sent to all active members of the association informing them of the purpose and nature of the study, providing assurances that none of the participants had been provided with details about the other participants in the study and that the management and collection data would be completely anonymous and would not be subjected to any risk.

The raw data collected from the survey were stored in a database on a single computer protected by the co-author’s password [12].

### 3.3. Statistical Analysis

Participants were asked to rate their level of agreement regarding whether they considered items in the survey to be a facilitator/barrier to palliative care.

Responses to all statements were captured in a four-level Likert scale, considering the score 1 as “Strongly disagree” and 4 as “strongly agree”.

The decision was made to include an even number for the response radius to avoid the possibility of choosing the central value (corresponding to a neutral opinion, known as the “median effect”). To determine the composition of the groups of participants resulting from the data in which they aggregated based on their responses to the 73 items, latent class analysis (LCA) was used, evaluating the suitability of 5 models (from 2 to 6 classes). We used several statistical fit indices to select the optimal number of latent classes based on what was proposed by Shevlin et al. [25]. We used the likelihood ratio χ2 and three goodness-of-fit measures to explore the adequacy of model fit and to compare competing models. This included the Akaike information criterion [26], the Bayesian information criterion [27] and the sample size-adjusted BIC [28]. The multinomial logistic regression technique was used to evaluate the association between class membership and demographic and clinical variables. Finally, odds ratios were used to determine the probability of obtaining a score on a given variable compared to the control group in terms of increase or decrease.

Data were coded and analyzed using the Statistical Package for Social Sciences (SPSS) ver. 22; LCA was conducted with R software. All tests were two-tailed (α = 0.05).

## 4. Results

There were 205 respondents in total, with a participation rate of 33%. Most respondents were women (85.5%); the mean age was 47.7 years. They had an average of 25 years of clinical experience and 20 years in nephrology units; most worked in public hospitals (70.5%). A total of 24.4% were atheists, and of those who acknowledged their religion, most were Catholic (71.4%) (Table 1).

In the Supplementary Material, we report the percentage of agreement for each item considered.

### Latent Class Analysis

**Figure 1:**
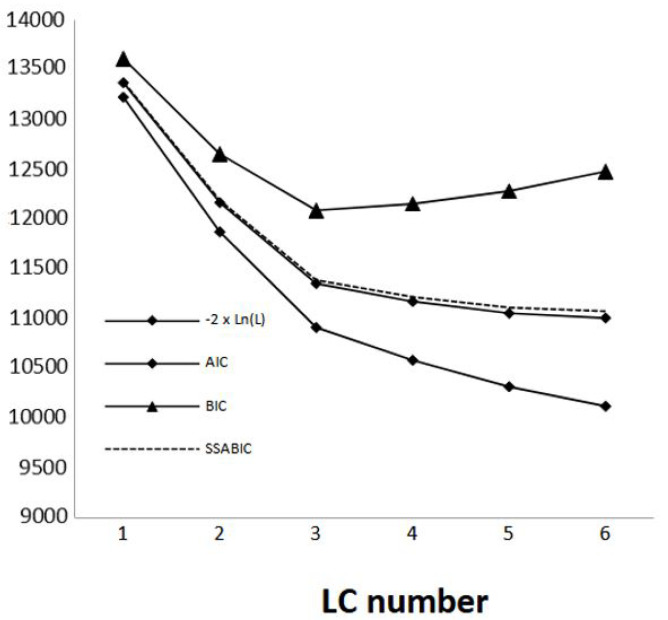
LCA fit indices for the total items: *y*-axis reports the value of each of the index scores considered; *x*-axis reports the number of latent class solutions. The best solution is generally the solution with lowest index values. 2ln(L) = likelihood ratio; AIC = Akaike information criterion; BIC = Bayesian information criterion; SSABIC = sample-size adjusted BIC.

The AIC and the SSABIC were lower in the three-class model than in the two-class model, and they decreased for higher-number class models, while the BIC reached the minimum value with the three-class model. Therefore, we selected the three-class model as the optimal solution (Figure 1).

**Figure 2:**
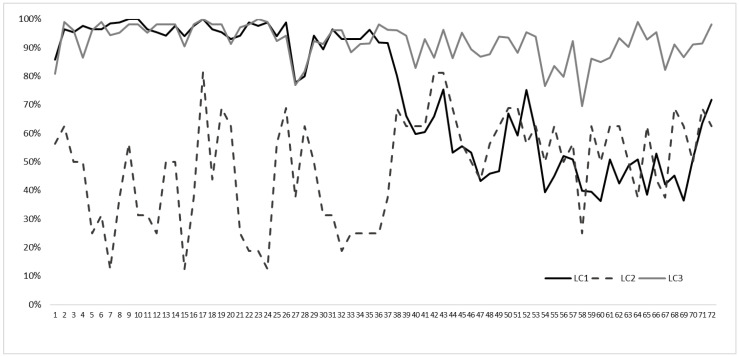
Profile plots for the 3-class solution. On the *y*-axis, class-specific mean scores as proportions of the maximum score. On the *x*-axis, the 73 items.

In the three-class solution, class 1 (LC1) included 85 individuals (41.7%) (Figure 2, black continuous line); it was characterized by the highest agreement percentages of facilitators (items 1–38). For all items, the endorsement rate was larger than 90%, except item 1 (Impartial listening), item 27 (Medical staff have palliative care experience) and item 28 (Nursing staff have palliative care experience), where the percentage of agreement was approximately 80%; on the contrary, in LC1, the percentage of agreement with the statements relating to barriers was much lower, with an average value of 53.7%.

LC2 included only 16 individuals (7.8%) (Figure 2, black dashed line), and it was characterized by a lower endorsement of facilitators, with a mean agreement of 39%; moreover, the highest heterogeneity among facilitators was recorded, unlike the other two classes (S.D. = 18.1%). A lower endorsement was reported for item 7 (Adequate education on approaching end-of-life by medical staff), item 15 (Patients talking about approaching end-of-life), items 22–24 (Providing post-registration training to nephrology nurses; Medical staff communicating effectively; Collaboration with a palliative care team in the community; respectively) and item 32 (Availability of psychological support in complex communication). Interestingly, the percentage of agreement for statements relating to barriers was similar to the LC1 profile. Finally, class 3 (LC3) included 144 individuals (50.5%), (Figure 2, grey continuous line) and was characterized by a relatively high probability (>80%) of item endorsement irrespective of content and barriers and facilitators.

Using multinomial regression, the predictive variables significantly associated with LC partnership have been calculated (−2 × log_likelihood = 286.4; Likelihood Ratio Tests X^2^_14_ = 38.3; *p* < 0.001); for regression models with a categorical dependent variable, it was not possible to compute a single R^2^ statistic that had all of the characteristics of R^2^ in the linear regression model, so these approximations are computed instead. Then, R^2^ Nagelkerke (its range is 0–1, R^2^ in linear regression) was calculated with a value of 0.223. At the end of the forward stepwise elimination process, only two variables were significantly associated with LC membership: “Years in nephrology” (X^2^_2_ = 7.02; *p* = 0.03) and “macro geographic area” (Tests X^2^_12_ = 33.4; *p* = 0.001). LC1 had been considered baseline; LC2 was not significantly different from LC1, whilst LC3 showed odd ratios (O.R.) significantly different from the expected value. In particular, “years of clinical practice” variables showed O.R. = 0.97 (95% C.I. = 0.94–0.99; *p* = 0.042), “Central Europe” (O.R. = 4.19; 95% C.I. = 1.33–13.17; *p* = 0.014) and “Eastern Europe” (O.R. = 4.18; 95% C.I. = 1.46–11.91; *p* = 0.007) demonstrated a significant O.R. also. In summary, the subjects belonging to the LC had less experience in nephrology than those in LC1 and were more likely to live in Central and Eastern Europe.

## 5. Discussion

From the statistical analysis, three classes of subjects emerged. The answers provided by the first class, LC1, were comparable to those of the third class, LC3, with regard to facilitators, and the total number of respondents exceeded 90% of the participants. In particular, some items of strong agreement, such as n. 11, 17 and 22, focused on respecting end-of-life care, the dignity given to the patient and following best practices. This appeared to be in line with what was reported by Young [13] and confirmed in the Delphi study by de Barbieri [12] in which even the experts believed that “advanced care planning” based on best practices and adequate communication with the patient was essential. However, these two groups had a strong disagreement in items 1, 27 and 28 (impartial listening, medical and nursing staff with palliative care experience). These appeared controversial and in contrast with the fundamental values of health professionals which, as reported by Fasset et al. [29], had a clear impact on the integration of PC in the management of patients approaching the end of life. In fact, no personal beliefs and values should influence the nurse’s attitude to listening and caring [17]. It therefore becomes clear that a healthcare approach focused primarily on alleviating physical pain is no longer adequate and that responding to people with palliative care needs inevitably requires the involvement of the spiritual dimension of each person. People with advanced illnesses openly express the importance of their spiritual needs being recognized and addressed by healthcare professionals, from a simple act of kindness to an empathetic connection with their suffering [30].

Regarding the “years in nephrology”, already in 2008, Ceccarelli [31] considered the introduction of “advanced care planning” to be essential to counter the phenomenon of lack of information and experience in this field; the two phenomena, in fact, have always been presented coupled, as well as in the Delphi study [12], which presented them with great internal stability on medium/low values of adherence. Here, however, both groups reported greater disagreement in the experience factor rather than education. It may be that over time the awareness of the issue of PC has been addressed at the level of university courses but has not been adequately responded to in the clinical arena. This was confirmed by an American study which reported that it is difficult to initiate discussion of the issue regarding the end of life despite the ability to identify patients with advanced kidney disease with a high probability of death, in particular regarding the suspension of dialysis; a discussion is therefore often avoided [32].

The responses of the two groups regarding barriers to PC were clearly distinguished; LC3 had a relatively high degree of agreement while LC1 was lower. It may be that LC1 did not consider the barriers as important or present in their working reality, or with their enhanced experience, they were able to face the obstacles that arose. The latter hypothesis was confirmed by the multinomial regression, which indicated an older group of respondents in LC1, with more work experience.

The group that differed regarding the facilitators was LC2, which reported high levels of disagreement, particularly in the items relating to communication and the approach to the end of life. However, the scarcity of respondents in this group led to no significant association, as reported by multinomial regression.

It is interesting to note how the barriers between LC1 and LC2 were almost overlapping, while LC3 clearly differed with a high level of agreement with respect to the items proposed. Using multinomial regression, we were able to identify a geographic provenience of LC3 in the countries of Eastern and Central Europe. This high level of agreement identified greater difficulties in managing PC in those countries in line with the literature. Clark [33] reported on the difficulties inherent in this geographical area in supporting an appropriate network of PC and humanization of care.

The four most significant obstacles to the development of PC in this geographical area are the limited financial and material resources, the problems relating to the availability of opioids, the lack of public awareness and recognition of the topic of palliative care even as a specialization and, finally, the lack of education and training programs on the topic.

An enormous and growing need to address the topic of palliative care is spreading across the world [34]. It is estimated that the need for palliative care will double between now and 2060, especially in low-income countries, among older adults and people with dementia [34]. The need for global action to integrate palliative care into health policies and systems is now common, but there continues to be a lack of political recognition, investment and supporting research evidence. The progress to achieve this goal is still slow, and it is not clear whether political interventions can increase the speed and volume of the development of palliative care worldwide; however, it is desirable to increase awareness and have maneuvering power in not only the health sector but also the social sector [33].

As emerged from the answers of participants to the questionnaire, the development of PC practices in many countries continues to remain uncoordinated, probably due to limited and poor investments and the limited capacity of services provided [33], as described above.

Finally, it is important to underline that only the years of experience and geographic origin variables had statistical significance at a predictive level of giving a score of agreement, while other variables (gender, working organization and religion) did not have an impact.

### 5.1. Limitations

The limitations of the study include the fact that the EDTNA/ERCA respondents did not equally represent all European countries. Some participants had more senior management positions, which may have impacted their responses, as they were likely to have had reduced clinical contact directly with patients.

A limitation of the study is represented by the fact that the survey was sent exclusively in English, which probably reduced the participation rate of participants compared to providing the survey translated into their own language.

This study, furthermore, only included nurses, and other HCPs were excluded. Further studies should include the views of medical and allied healthcare staff.

Limitations also include the lack of stimulation provided by face-to-face conference meetings [35], which may have contributed to the lack of response from some members invited to the questionnaire.

### 5.2. Implications for Clinical Practice

This questionnaire can be a starting point for investigating the role of PC in people with ESKD at a wider European or international level, to explore the experiences of providing PC in dialysis and, in particular, to confirm the correlation with years of experience and geographical area. The degree of adherence to the statements allows us, at first glance, to understand the different cultures on the topic and the approach used. Although it was born and developed in a predominantly European context, the theme and statements are well suited to different countries and cultures and can be studied as a subdivision in the future, thus allowing the priorities of one country to be identified compared to another.

An interesting proposal for future studies could be to submit or create new statements to administer directly to patients and caregivers, as it would be useful to know the direct point of view on the topic of palliative care and compare the visions with those of healthcare workers.

Finally, to validate the findings and explore the identified facilitators and barriers in more depth, longitudinal studies or qualitative research could be conducted.

## 6. Conclusions

This study demonstrates that there are some variables that have an impact on facilities or barriers in assisting patients in PC in dialysis units, such as the geographical location and years of experience of nursing staff. Some were able to deal with complex PC-related situations and PC was well embedded in the care pathway, while others considered barriers that presented as obstacles to the implementation of adequate treatment.

Greater experience led to agreement with facilitators and disagreement with many barriers presented, while the geographical area of the nurses particularly impacted the scores related to the barriers in the context of a greater difficulty to implement PC (as seen in some countries of Eastern and Central Europe).

## Figures and Tables

**Table 1:** Descriptive statistics were reported as counts (N) and percentages (%) for categorical variables; mean and standard deviation (S.D.) for measures.

		N	%
**Gender**	Male	28	14.5%
	Female	161	85.5%
**Geographical Area**	Central Europe	35	17.1%
	Eastern Europe	58	28.3%
	Southern Europe	66	32.2%
	Northern Europe	15	7.3%
	Non-European countries	31	15.1%
**Working Organization**	Public	136	70.5%
	Private	57	29.5%
**Practicing**	Unbeliever	50	24.4%
	Non-practicing believer	64	31.2%
	Practicing believer	91	44.4%
**Religion**	Christian Catholic	120	71.4%
	Christian Orthodox	19	11.3%
	Christian Protestant	25	14.9%
	Islamic	4	2.4%
		**Mean**	**S.D.**
**Age**		47.7	9.8
**Years of overall clinical practice**		25.2	10.5
**Years of clinical practice in nephrology**		20.1	10.1

## Data Availability

The data presented in this study are available on request from the corresponding author.

## Supplementary Materials

The following supporting information can be downloaded at: https://www.mdpi.com/article/10.3390/nursrep14010018/s1, Table S1. List of item.

## Public Involvement Statement

There was no public involvement in any aspect of this research.

## Guidelines and Standards Statement

This manuscript was drafted in accordance with the STROBE guidelines for observational research.
