# Supplementary material for: Facilitators and Barriers to Receiving Palliative Care in People with Kidney Disease: Predictive Factors from an International Nursing Perspective
Source: Nurs Rep. 2024 Jan 22;14(1):220–9. doi: 10.3390/nursrep14010018 (PMC10885022; doi:10.3390/nursrep14010018)
Supplement: Supplementary file 1 [file nursrep-14-00018-s001.zip › nursrep-2695177-supplementary.pdf]

Supplementary Materials [12]

N	Item	%
1	Impartial listening	81:0%
2	Active listening	95,1%
3	Communicating truthfully and clearly about patients’ prognosis	92,2%
4	Involving the family of the patient in choosing dialysis or palliative care	88,3%
5	A collaborative approach between renal services in hospital and the community	90,7%
6	Support of a Renal Palliative Care team	92,7%
7	Adequate education on approaching end-of-life by medical staff	89,8%
8	Adequate education on approaching end-of-life by nursing staff	92,2%
9	Reassuring patients that they will not feel abandoned if they choose palliative care	95,6%
10	Presence of a multiprofessional team	93,7%
11	An enviroment that supports innovation, research, education and dissemination of best practices	90,7%
12	A focus on symptom management and psychosocial support	91,2%
13	Patient able to die in a place of their choice	92,7%
14	Good management of symptoms	94,1%
15	Patients talking about approaching end-of-life	85,9%
16	Presence of a specific plan of care which includes advanced care planning	93,2%
17	Treating the dying patient with dignity and respect	98,5%
1	End of life care competencies for medical staff included in university curricula	93,2%

1

8		
19	End of life care competencies for nursing staff included in university curricula	94,6%
20	Participation of family/carers in decision-making	89,8%
21	Presence of national guidelines that support clinical practice in the end of life period	90,2%
22	Providing post-registration training to nephrology nurses	92,2%
23	Providing a stimulating work enviroment with places where teams can meet, interact and reflect	92,7%
24	Collaboration with a palliative care team in the community	92,2%
25	Medical staff communicating effectively	90,2%
26	Nursing staff communicating effectively	94,1%
27	Medical staff have palliative care experience	74,1%
28	Nursing staff have palliative care experience	79,5%
29	Presence in the hospital of a positive attitude towards palliative care	89,8%
30	Implementation of standard scales for symptom assessment	85,9%
31	Information for the family/carers about the protection and promotion of life until death while receiving palliative care	91,2%
32	Availibility of psychological support in complex communication	88,8%
3	Presence of standard hospital procedures for palliative care	85,4%

33		
34	Presence of a network of nursing home staff and residential care home staff	86,8%
35	Identification of cultural barriers among healthcare professionals that could prevent uptake of palliative care.	88,3%
36	Knowledge about the different cultural approaches to the end of life	89,8%
37	Knowledge about spiritual needs in the end of life period	89,8%
38	Lack of training and resources to conduct difficult discussions about deterioration	87,3%
39	Lack of time to conduct difficult discussions about deterioration	80,0%
40	Involving family/carer in end of life decision making	71,7%
41	Lack of collaboration between nursing and medical staff	77,1%
42	Refusal by patient to accept deterioration and approaching death	77,6%
43	Refusal by family to accept deterioration and approaching death of patient	86,3%
44	Fear of staff to family reactions to palliative care	71,2%
45	Feel unprepared to start difficult conversations, and having a fear of using the wrong words	75,6%
46	Cultural beliefs and practices	71,2%
47	Spiritual beliefs	65,4%
4	A lack of knowledge about which patients will benefit from renal replacement therapy	67,8%

8	rather than palliative care	
49	Individual survival and quality of life predictions difficult in the elderly with end-Stage Kidney Disease	71,7%
50	Absence of adequate palliative care services in rural areas	80,5%
51	The patient and the patient's family think that withdrawing from dialysis is the same as euthanasia	74,6%
52	Nephrologists focus on biomedical factors and have an inherent instinct to prolong life	83,9%
53	Nephrologists try and mantain hope for the future	77,6%
54	Regret in patient and family about stopping dialysis	59,0%
55	Limited evidence to support renal palliative care in the literature	65,9%
56	Family/carer's involvement in the decision-making process	65,9%
57	Clinicians influencing the patient to make a particular decision	72,2%
58	Nurses influencing the patient to make a particular decision	53,7%
59	Shared treatment decision-making is not a common term in the renal unit	64,9%
60	End-of-life discussions are often not started by the health care team	62,0%
61	Difficulty in estimating prognosis	69,8%
62	Death considered a taboo subject	69,8%
6	Nurses lack of experience conducting palliative care	69,8%

3		
64	Medical staff lack of experience conducting palliative care	74,1%
65	Beliefs in preservation of hope and life	67,8%
66	Medical staff lack experience in end of life care	73,7%
67	Nurses lack experience in end of life care	62,0%
68	Worries about legal consequences	70,2%
69	Prolonging life viewed as more important than honoring a patient's request to forgo life-sustaining treatment	63,9%
70	Family disagrees with the patient's wishes	71,2%
71	Insufficient information about palliative care in nursing university curriculum	78,0%
72	Insufficient information about palliative care during medical training	84,4%
73	Stigma of palliative care in some cultures as an acceptance of death	79,5%

Percentage of statement of 73 items considered.
